# Small intestinal perforation caused by folded polyethylene drug‐wrapping film

**DOI:** 10.1002/jgf2.421

**Published:** 2021-01-31

**Authors:** Sayako Maeda, Koji Takaori

**Affiliations:** ^1^ Division of Nephrology Department of Internal Medicine Japanese Red Cross Otsu Hospital Otsu Japan

**Keywords:** polyethylene drug‐wrapping film, intestinal perforations

## Abstract

An 81‐year‐old female patient underwent an emergent surgery because of small intestinal perforation. Within a 5‐mm‐sized hole in ileum, a half‐folded, empty polyethylene drug‐wrapping film with the drug names, date, and her name was found.

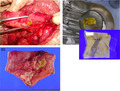

An 81‐year‐old female patient was admitted to our hospital because of urinary tract infection due to urinary retention, which improved with urethral catheterization and sulbactam sodium and ampicillin sodium. A day prior to discharge, the patient complained of abdominal pain, diarrhea, and a fever of 38.0℃.

The patient reported a history of undergoing peritoneal dialysis 5 years ago for a duration of 5 months because of end‐stage renal disease due to diabetic nephropathy and subsequent hemodialysis three times a week for 4 years. About 1 year ago, she had uterine sutures placed because of perforative peritonitis due to pyometra with uterine perforation.

Her blood pressure was 130/60 mmHg, and body temperature was 36.7℃. Her abdomen was hard with rebound tenderness. Laboratory data demonstrated a white blood cell count of 12 300/µL, hemoglobin 11.4 g/dL, platelet count 339 000/µL, CRP 22.1 mg/dL, serum creatinine 3.1 mg/dL, and BUN 23.5 mg/dL.

Her computed tomography revealed a rupture in the wall of the small intestine and fluid collection in the abdominal cavity underneath it, suggesting small intestinal perforation (Figure 1). She underwent an emergent surgery during which the surgeons found a 5‐mm‐sized hole about 100 cm from the ileocecal region (Figure 2A), and, within it just near the hole, a half‐folded, empty polyethylene wrapping film was found (Figure 2B). As she had severe adhesions in the abdominal cavity, 20 cm of her ileum around the hole was excised. The resected ileum revealed a layer of pus and infiltration of neutrophils in the serous membrane and a layer of tissue under the serosa, consistent with peritonitis (Figure 2C). The patient completely recovered following the operation.

**FIGURE 1:**
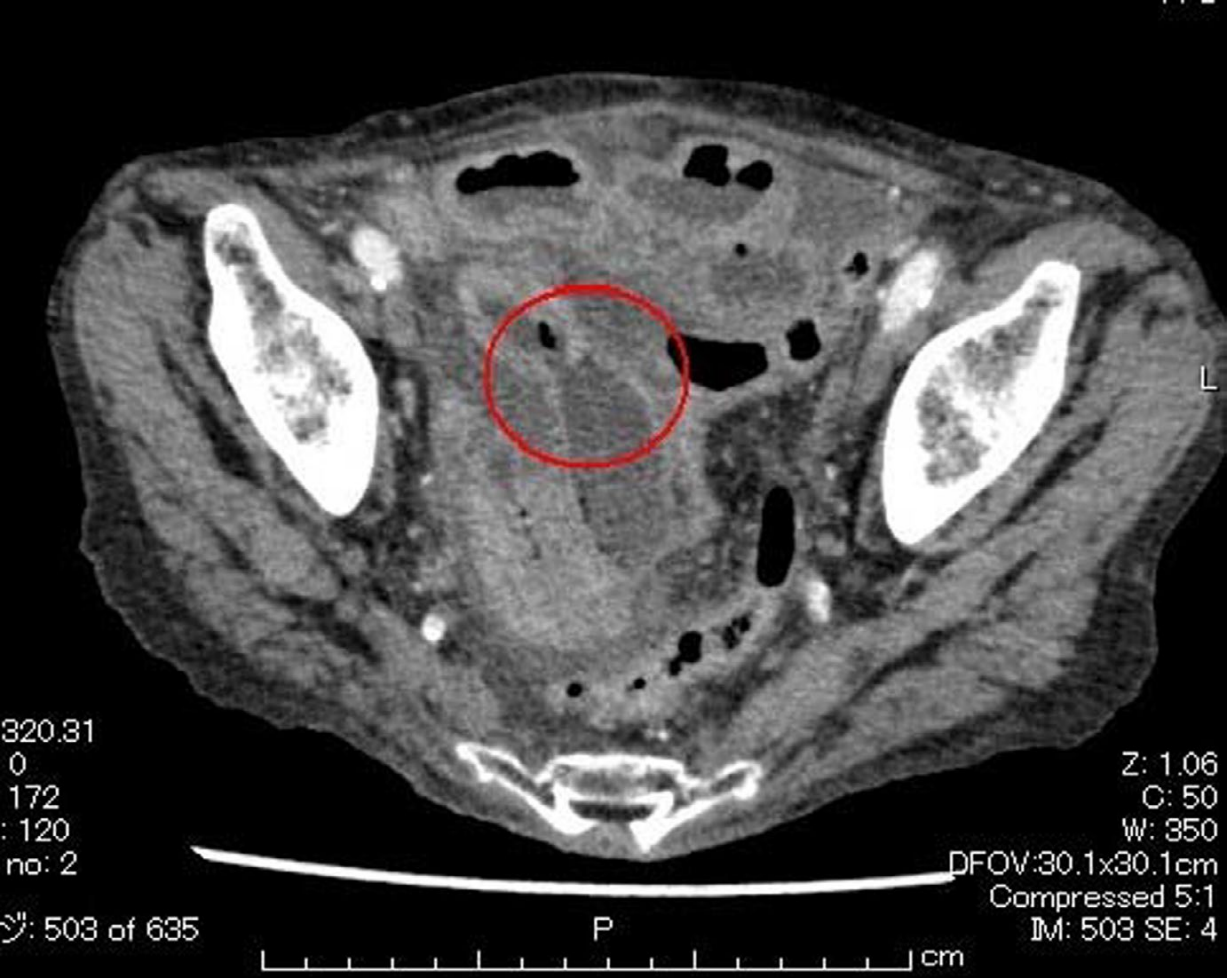
A rupture in the wall of the small intestine and fluid collection in the abdominal cavity underneath it, suggesting small intestinal perforation

**FIGURE 2:**
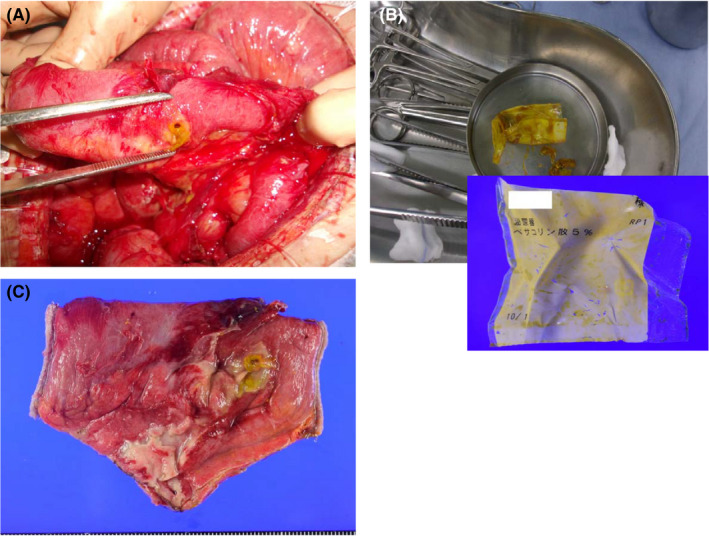
A 5‐mm‐sized hole was found approximately 100 cm from the ileocecal region (A), and a half‐folded, empty polyethylene wrapping film was found within the hole (B). The resected ileum revealed a layer of pus (C)

Of note, the pharmacists place all tablets and powders together in a clear film. A few days before the operation, while the patient was sound asleep, her nurse had cut one side of the film package and left it on her bedside. When the patient, who had mild dementia and poor eyesight, woke up, she found the film package, folded it into a smaller size, and swallowed it, mistaking it for her prepared package wrapped by a self‐dissolving wafer paper. The nurse remembered that a few days prior to the operation, the patient had complained about hardness of the wrapping film of the drugs.

Polyethylene drug‐wrapping film is thin and soft, but when folded, the corners are hard and sharp, which could be stuck in a narrow space in the intestinal cavity with severe adhesions after prior perforative peritonitis. Moreover, she had obstinate constipation.

Several cases of intestinal perforations due to aspiration of press through package (PTP) have been reported^1^, ^2^, ^3^, ^4^; however, corners of the folded polyethylene drug‐wrapping film could also injure the intestine. Therefore, special care should be taken for old patients to avoid unguarded ingestion of not only PTP but also clear polyethylene drug‐wrapping film. To prevent the accidental drug‐wrapping film ingestion by elderly people with dementia, developing a safe “dementia‐resistant package” is expected and desirable, similar to the “child‐resistant package” for children^5^.

## CONFLICT OF INTEREST

The authors have stated explicitly that there are no conflicts of interest in connection with this article.

## INFORMED CONSENT

Patient consent was obtained, and patient anonymity was preserved.
